# An Empirical Model for the Churning Losses Prediction of Fluid Flow Analysis in Axial Piston Pumps

**DOI:** 10.3390/mi12040398

**Published:** 2021-04-03

**Authors:** Ying Li, Xing Chen, Hao Luo, Jin Zhang

**Affiliations:** 1 School of Mechanical Engineering, Yanshan University, Qinhuangdao 066004, China; yingli@ysu.edu.cn (Y.L.); chenxing@stumail.ysu.edu.cn (X.C.); luohaolh@stumail.ysu.edu.cn (H.L.); 2 State Key Laboratory of Fluid Power and Mechatronic Systems, Zhejiang University, Hangzhou 310027, China

**Keywords:** axial piston pumps, churning losses, prediction model

## Abstract

The manufacturing development of axial piston pumps usually takes the trend of high speed and miniaturization, and increases power density. Axial piston pumps are usually characterized as high speed to improve the power density; thus, high-speed churning losses caused by the internal rotating components stirring the oil can increase significantly. In order to improve the efficiency, more attention should be given to the churning losses in axial piston pumps, especially in high-speed conditions. Using the method of least-squares curve fitting, this paper establishes a series of formulas based on the churning losses test rig over a wide range of speeds, which enable accurate predictions of churning losses on the cylinder block and pistons. The reduction coefficient of flow resistance of multi-pistons as calculated. The new churning losses formula devoted to the cylinder block and rotating pistons was validated by comparison with experimental evidence in different geometries of axial piston pumps. According to the prediction model of churning losses, some valuable guidance methods are proposed to reduce the energy losses of the axial piston pump, which are the theoretical support for the miniaturization of axial piston pump manufacturing.

## 1. Introduction

**Figure 1:**
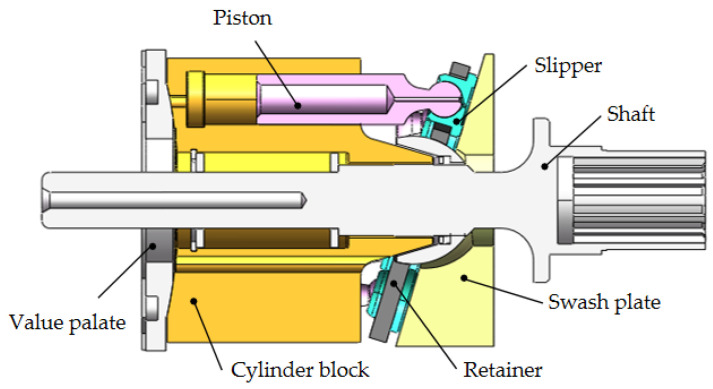
Configuration of a typical axial piston pump.

The axial piston pump is an important power source in hydraulic systems that is widely used in various occasions, such as engineering machinery and airplanes [1,2,3], because axial piston-type hydraulic pumps achieve high-power densities and a long service life. Figure 1 shows a typical configuration of an axial piston pump, which consists of three main rotating components: a cylinder block, pistons and slippers. The non-rotating parts are the valve plate, retainer and swash plate. The main rotating components are driven by the shaft. The pistons are arranged on the cylinder block bores, and piston heads are mounted in the slippers. When the motor drives the transmission shaft to rotate, the cylinder block and the pistons rotate together. The piston heads always keep contact with the swash plate under the action of the return mechanism. Because the swash plate and the cylinder block are at an angle, the pistons reciprocate in the cylinder block bores. The pump sucks oil when the pistons extend, and discharges oil through a valve plate.

However, continuously increasing power densities requires the development of accurate methods of estimating the efficiency of axial piston pumps in order to prevent overheating, failures and fault diagnoses. Efficiency and related thermal consequences have therefore become significant issues which have to be predicted and taken into consideration in the design stage of axial piston pumps [4,5]. It is well known that, for low-to-medium speed axial piston pumps, power losses are mainly caused by friction and leakage in three friction pairs (swash plate/slipper pair, piston/cylinder block pair and cylinder block/valve plate pair). Whereas, for high-speed applications, churning losses caused by the main rotating parts become prominent in the power losses of axial piston pumps.

Focusing on churning, Jang [6] first proposed the concept of churning losses, which is composed of the cylinder block and piston/slipper units. Xu et al. [3,7] investigated the effects of the rotating cylinder block and circling pistons on churning losses in axial piston pumps, respectively. The proportion of the rotating cylinder block and circling pistons on churning losses was measured. The research results showed that the mathematical model of the churning losses based on the assumption of laminar flow is not suitable for high-speed working conditions. Moreover, the rotating cylinder block is the main factor affecting the churning losses at high rotation speeds. Murrenhoff et al. [8,9] performed the simplified model of churning losses to improve the efficiency on the basis of the laminar and empirical drag coefficient of single-piston assumption; they also established and verified the CFD (computational fluid dynamics) simulation model of churning losses in axial piston pumps. Ivantysynova and Shang et al. [5,10] derived the energy dissipation equation for the cylinder block and pistons from the Navier–Stokes equation to evaluate the churning losses in low-speed axial piston pumps. They also calculated and then measured the case and outlet port temperatures by taking the churning losses into consideration. The churning losses and total losses under various operating conditions were measured. The percentage of churning losses in total power losses was different under different operating conditions. The churning losses accounted for about 10% of the total power losses under 3000 rpm. Rahmfeldet et al. [11,12] pointed out that a further option to improve hydrostatic unit efficiency was the elimination of churning losses, preferably in hydrostatic bent-axis motors due to their high speeds. They extended the mathematical model for churning losses, which considered the shaft and synchronous joint. They also verified this churning loss model through CFD simulations in bent-axis piston motors. Recently, Jing et al. [13] conducted experimental research on the churning losses of the pump operating at low speeds in both dry and wet casing conditions, and discussed the importance of the oil fill level in an axial piston pump. Moslåttet et al. [14] provided a new model of total torque losses in variable displacement axial piston motors. The churning losses caused by the piston/slipper units were included in the expression of the total torque losses. The churning losses were given under 2000 rpm in an axial piston motor. Huang et al. [15,16,17] presented a novel 2D high-speed piston pump. They used analytical and numerical methods to study the churning losses caused by the rotor and proposed that, especially when the rotational speed is above 8000 rpm, the considerations of turbulence and cavitation need to be added to both the mathematical models and CFD simulations.

From a thorough review of the literature, it appears that the churning losses model for axial piston pumps plays a critical role in improving efficiency and predicting overheating. It seems that all the mathematical models for the churning losses mentioned above were based on the laminar and empirical drag coefficient of single-piston assumptions at a low speed. However, the laminar and empirical drag coefficient of single-piston assumptions is not suitable for high-speed axial piston pumps, because the Reynolds number increases significantly with the increasing speed and a reduction coefficient exists in the flow resistance around the multi-pistons. A reliable and accurate high-speed churning loss model remains to be researched and developed.

Despite the fact that the mathematical model of churning losses at high speed has not been studied, churning losses have been investigated in external gear machines and geared transmissions. Vacca [18,19] proposed the expression for torque losses in external gear machines. Changenet et al. [20] studied various fluid flow states generated by a pinion running that was partly immersed in an oil bath in geared transmissions. They also established a loss formula under different flow states in geared transmissions. Theissen et al. [8] found that a very simple measure to reduce agitation losses was to use a lower oil viscosity. These studies on the external gear machines and geared transmissions have significance in guiding the study of axial piston pumps.

The goal of this paper is to derive the mathematical model for the churning losses of the axial piston pumps in high-speed conditions using the differential analysis of least-squares curve fitting. The reduction coefficient in the flow resistance around the multi-pistons is then evaluated on the basis of the experimental results. The churning losses caused by the rotating cylinder block and pistons were respectively measured. The results of churning losses under different working conditions were tested on the test rig, and the effectiveness of the model was verified. The results can establish more comprehensive and accurate theoretical models for estimating the churning losses of axial piston pumps in high-speed conditions. It can also provide guidance for reducing churning losses, especially in high-speed conditions.

## 2. Materials and Methods

**Figure 2:**
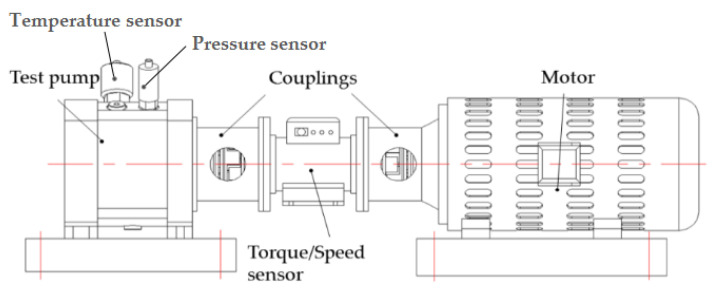
Test rig of churning losses.

**Figure 3:**
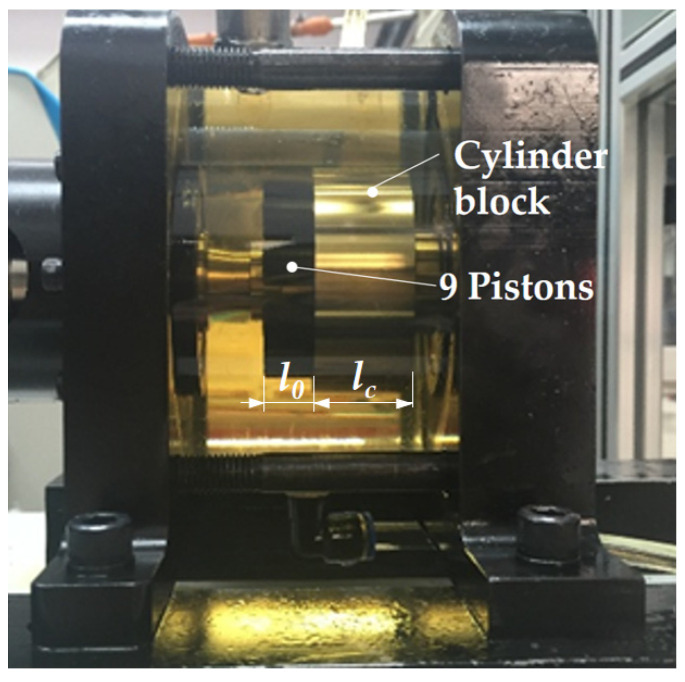
Oil flow around the pistons and cylinder block.

The specific test rig (Figure 2) described by the authors of [3] was used to quantify the churning losses. The test pump was designed on the basis of a high-speed axial piston pump prototype used for aircrafts. A motor operated the shaft rotational speeds up to a maximum value of 16,000 rpm. The casing of the test pump was made of Plexiglas for the purpose of obtaining the flow state around the pistons and cylinder block, as shown in Figure 3. The test pump only has a piston/cylinder pair. The slippers and swash plate were removed because of the friction of the slippers on the swash plate and the friction of the piston/cylinder block pair. We measured the subtraction of the churning losses torque acting on the shaft by the cylinder block rotating alone to obtain the churning losses torque due to the rotating cylinder block. The churning losses due to the pistons were calculated by the subtraction of churning losses of the piston/cylinder and cylinder block. These frictions had a higher influence on the measured torque. The difference of the flow pattern without the slippers and swash plate was small, because the swash plate is not a rotating part and the lengths of slippers are smaller than the pistons. Churning losses produced by the circling pistons significantly depend on the dimensions and rotational motion of the pistons. Moreover, the dimensions of the pistons in the test pump were as the same as those in the real pump in another study [21]. Therefore, the results of the losses produced by the circling pistons and cylinder block in this simplified setting can provide useful information in the case with the slippers and swash plate. The test pump was measured with oil in the casing and then again in the absence of any oil. Churning losses were determined from direct torque measurements by a torque/speed sensor with a full-scale range of 10 N·m and a precision of ± 0.02 N·m. As shown in Figure 2, in order to measure the temperature and pressure in the test pump, a temperature sensor and a pressure sensor were installed on the top of the test pump. The energy lost by stirring becomes heat, which raises the temperature of the oil in the casing. As the rotation speed increases, the heat generated by the churning losses increases, and the oil temperature rise phenomenon becomes more and more obvious. Temperature is not a direct factor affecting the churning losses, but the temperature change does change the viscosity of oil, and thus, affects the churning losses. The oil in the test was preheated and the temperature was maintained at 35 ± 1 °C and the oil pressure was stabilized at 1.01 ± 0.01 bar. To isolate the net contributions of churning losses, the additional drag torque of the bearings was experimentally determined and subtracted from global torque measurements.

In Reference [3], the ratio of the rotating cylinder and circling pistons on churning losses was also measured. Two empirical churning torque formulas were derived under the form:

(1) $$P_{\mathrm{cc}}=M_{\mathrm{cc}}\omega =\left(M_{\mathrm{wc}}-M_{\mathrm{dc}}\right)\omega ,$$

(2) $$P_{cp}=M_{\mathrm{cp}}\omega =\left(M_{\mathrm{wcp}}-M_{\mathrm{dcp}}-M_{wc}+M_{\mathrm{dc}}\right)\omega ,$$

where *M*_cc_ is the experimental churning losses torque due to the rotation of the cylinder, *M*_wc_ is the experimental torque acting on the shaft because of the rotation of the cylinder with oil in the case, *M*_dc_ is the experimental torque acting on the shaft because of the rotation of the cylinder without oil in the case, *ω* is the rotation angular velocity, *M*_cp_ is the experimental churning losses torque due to the circling pistons, *M*_wcp_ is the experimental torque acting on the shaft because of the circling pistons and the rotating cylinder with oil in the case and *M*_dcp_ is the experimental torque acting on the shaft because of the circling pistons and the rotating cylinder without oil in the case [3].

The geometric dimensions of the test pump were the same as a high-speed axial piston pump prototype in Reference [21]. The geometric dimensions and operating conditions of the test pump are also provided in Table 1.

## 3. Results

### 3.1. Churning Losses—Formulas for the Cylinder Block

According to the authors of [22], the theoretical model of churning losses due to the rotation cylinder block can be written as:

(3) Pcm=2πω3lcRc4ρRe

where *ω* is the rotation angular velocity, *l*_c_ is the length of the cylinder block, *R*_c_ is the radius of the cylinder block, *ρ* is the density of the hydraulic fluid and Re is the Reynolds number. The result of this equation is an approximation, valid only for small values of the gap between the cylinder block and the housing internal surface and laminar flows.

The Reynolds number in the above equation can be defined as:

(4) Re=ωRctρμ−1,

where *μ* is the dynamic viscosity of the hydraulic fluid and *t* is the gap between the cylinder block and the housing internal surface.

**Figure 4:**
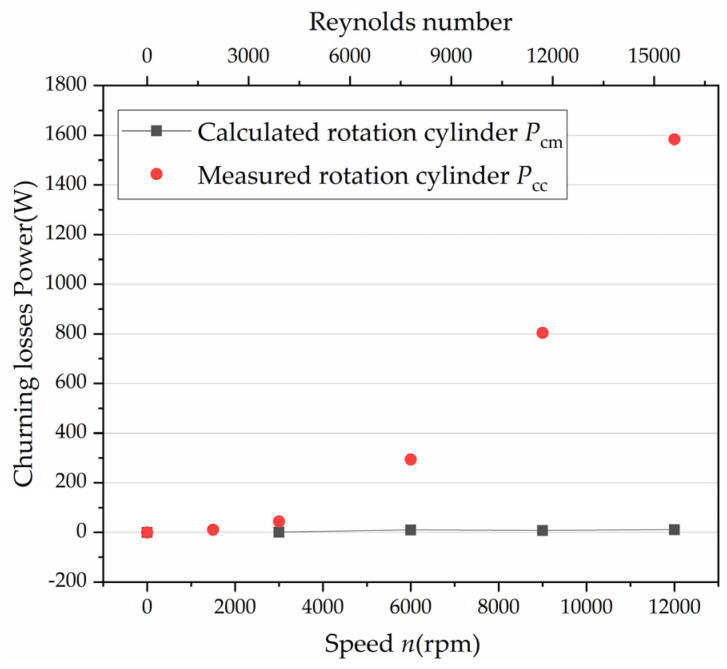
Churning losses: comparisons between the calculated and the experimental results.

**Figure 5:**
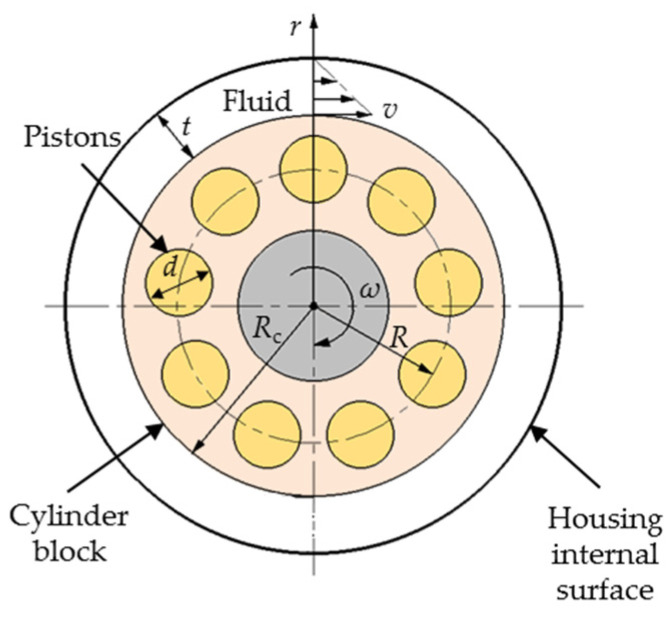
Churning losses due to the cylinder block: geometrical data.

Figure 4 shows the comparisons between the measured churning torques and the predictions using the above equations. It can be concluded that the laminar loss model for an axial piston pump shows good applicability at low speeds. More reliable formulas need to be established at high speeds. The former model may ascribe the failure of the inappropriate laminar flow assumption, which does not take the turbulent flow into consideration when the Reynolds number increases from 10^3^ to 10^4^ with the speed increasing from 3000 to 12,000 rpm, thus resulting in a poor correlation with the experimental data. Figure 5 shows the curve of the Reynolds number changing with the rotational speed.

The parameters affecting the churning losses were classified according to the type of influence and are described in Figure 5: the pump geometry was described by the radius of the cylinder block *R*_c_, length of the cylinder block *l*_c_ and the gap between the cylinder block and the housing internal surface *t*; the characteristics of the lubricant were described by the viscosity *μ* and density *ρ*; the working conditions were described by the rotational speed *ω*.

It was deduced that the churning losses power can be written as

(5) $$P_{\mathrm{cm}}=f(l_{c},R_{c},t,\rho (T),\mu (T),\omega ).$$

**Figure 6:**
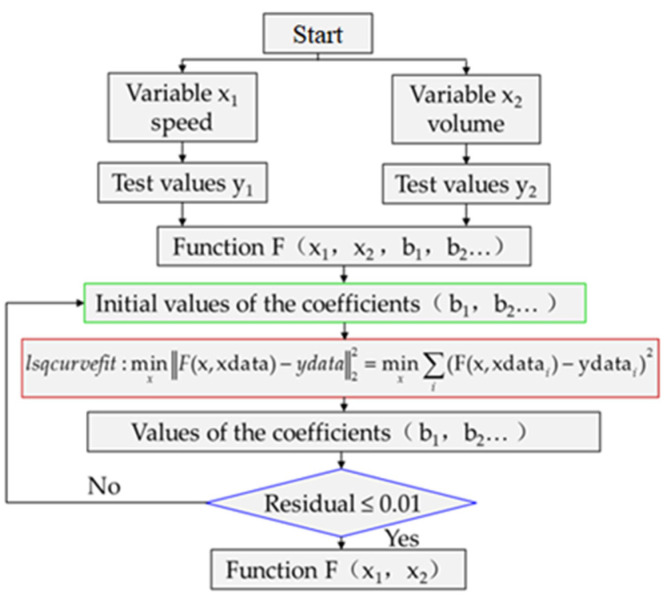
Method of least-squares curve fitting.

The factors affecting the churning power can be divided into three parts: the pump geometry, the properties of the oil and the operating conditions, as shown in Equation (5). The properties of oil used in the axial piston pump are usually determined according to the use requirements. In order to facilitate the analysis of control variables, the main factors affecting the churning power are reflected in the pump geometry and operating conditions, while the fluid parameters are the same. The churning losses are mainly related to the volume and speed of the oil involved in the churning. Thus, the speed and volume are defined as two variables and the corresponding test values can be obtained by the churning losses test rig. The function of the churning losses due to the cylinder block can be built through the initial values of the coefficients. Using the method of least-squares curve fitting, as shown in Figure 6, the residual can be calculated. If the residual is more than 0.01, the initial values of the coefficients should be assigned. The function of the churning losses due to the cylinder block with determined initial values of the coefficients can be obtained until the residual is less than 0.01.

The parameter identification was conducted on the basis of the procedure of least-squares curve fitting, thus leading to the expression of churning losses due to the cylinder block. The churning losses power *P_cm_*’ obtained by least-squares curve fitting is shown in Equation (6).

(6) $$P_{\mathrm{cm}}^{\text{’}}=\left(5.44\omega^{2}-973.57\omega \right)V+7.76\times 10^{-7}\omega^{3}-0.0021\omega^{2}+3.2\omega$$

It was found that churning loss increased with the increase of volume when 5.44*ω*^2^ − 973.57*ω* > 0 (*ω* > 178.97 rad/s). The high-speed turbulence pulsation and churning losses increased with the increase of the volume of oil involved in churning. Churning losses decreased with volume increase when 5.44*ω*^2^ − 973.57*ω* < 0 (*ω* < 178.97 rad/s). The low-speed viscous friction loss of laminar flow was dominant. The velocity gradient decreased with volume increase, and churning losses also decreased. Thus, Equation (6) can be rewritten as Equation (7), and the relationship between the churning loss and the rotational speed is shown as:

(7) $$P_{\mathrm{cm}}^{\text{’}}=7.76\times 10^{-7}\omega^{3}+\left(5.44V-0.0021\right)\omega^{2}+\left(3.2-973.57V\right)\omega .$$

The discriminant of Equation (7) can then be written as:

(8) $$\Delta =4\left[{\left(5.44V-0.0021\right)}^{2}-3\times 7.76\times 10^{-7}\left(3.2-973.57V\right)\right].$$

**Figure 7:**
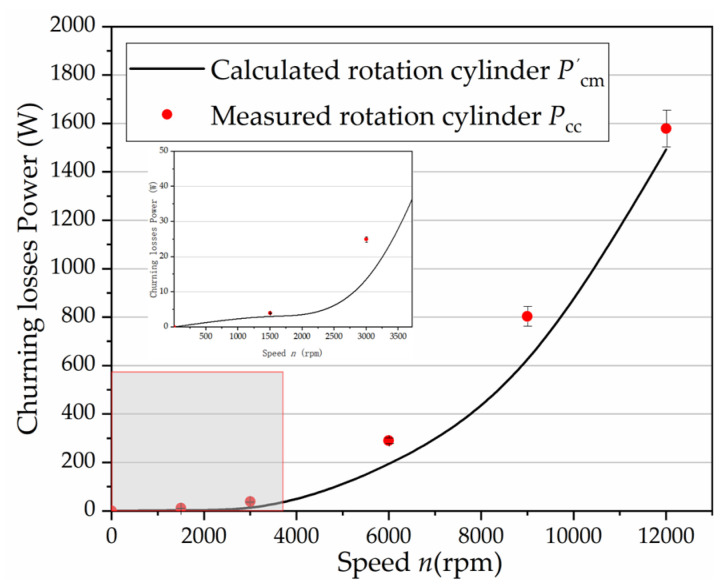
Comparisons between the experimental results and the results from the method of least-squares curve fitting for the cylinder block.

It was found that discriminant was less than 0 when *V* < 0.0008 m^3^. The oil volume in the high-speed pump housing was always less than 0.0008 m^3^, according to the structure size of the high-speed test pump, as shown in Table 1. The churning losses increased with the increase of rotating speed no matter how the volume changed in the high-speed axial piston pump. The results from the method of least-squares curve fitting for the cylinder block curve can be obtained as shown in Figure 7. The validity of the proposed formulation is also illustrated in Figure 7, in which the dots represent the experimental findings, while the solid lines account for the numerical results. These curves represent a sample of the results obtained from the repeated tests and simulations with various speeds in the 0–12,000 rpm range. A satisfactory agreement was observed in all cases.

### 3.2. Churning Losses—Formulas for Pistons

The theoretical model of churning losses due to the circling pistons can be written as:

(9) $$P_{\mathrm{pm}}=C_{d}\pi d\rho \omega^{3}R^{3}\sum_{i=1}^{z}\frac{\left[l_{0}-R\left(1-\cos(2\pi i/z)\right)\tan\gamma \right]}{2},$$

where *C*_d_ is the drag coefficient related to the Reynolds number, *d* is the diameter of pistons, *R* is the pitch circle radius of piston bores, *z* is the number of pistons, *l*_0_ is the length of the piston out of the cylinder at the outer dead point and *γ* is the swash plate angle [3]. This equation applies to laminar flow; therefore, it is not fit good at high Re.

**Figure 8:**
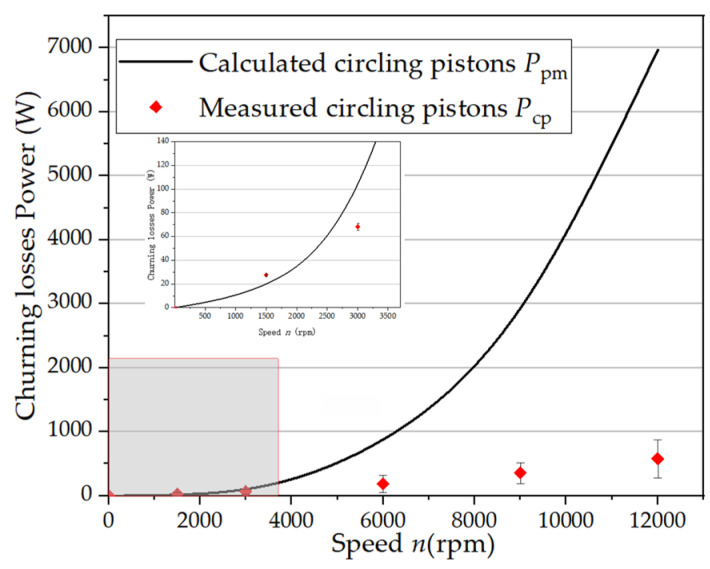
Churning losses: comparisons between the calculated and the experimental results.

Figure 8 shows a series of comparisons between the measured churning torques and the predictions using the above equation. It can be concluded that the above-mentioned theoretical model cannot be applied to the full range of rotation speed, which proves that the more reliable formula is reasonable. The circling flow of multi-pistons reduces the flow resistance between the pistons. The literature [23,24,25,26] shows that the resistance coefficient of the flow among multi-pistons has a reduced reduction coefficient compared with the resistance coefficient of a single-piston flow. Because of the existence of the reduction factor, the turbulence resistance coefficient *C*_d_ in formula 9 is affected, so the new turbulence resistance coefficient with the reduction coefficient should be ∑*C*_d_. Therefore, the measurement results of the churning losses are lower than the calculation results under the high-speed conditions.

According to the authors of [27], the reduction coefficient in the flow resistance around the multi-pistons can be defined as:

(10) $$k_{z}=\left(\frac{\sum C_{d}}{C_{d}}-1\right)/z,$$

where *k_z_* is the reduction coefficient, which is adjusted from the experimental results.

The difference between the measured circling pistons *P*_cm_ and the calculated circling pistons *P*_pm_ of formula 9 is the difference between *C*_d_ and ∑*C*_d_; therefore, the value of *k*_z_ is obtained from the measured value of experiment and formula 9. The equation means the difference between the ratio of the total drag coefficient of multi-pistons to the original single piston and 1.

The identified coefficient *k_z_* is listed in Table 2 with the range of 1500–12,000 rpm. The reduction coefficient was obviously decreased, and even negative, with the increase of the rotational speed, as shown in Table 2. It indicates that the effect of multi-piston circling was enhanced with the increasing speed.

**Figure 9:**
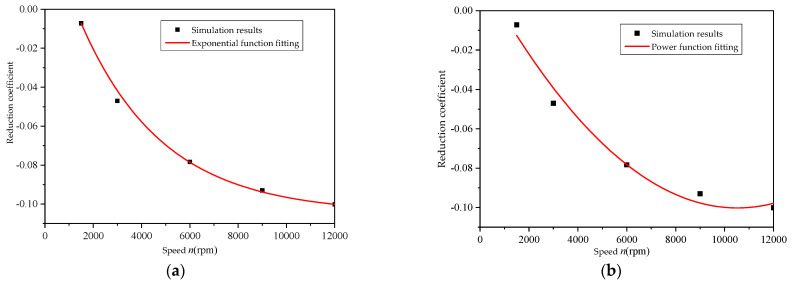
The variation law of the reduction coefficient with rotational speed: (**a**) exponential function fitting and (**b**) power function fitting.

**Figure 10:**
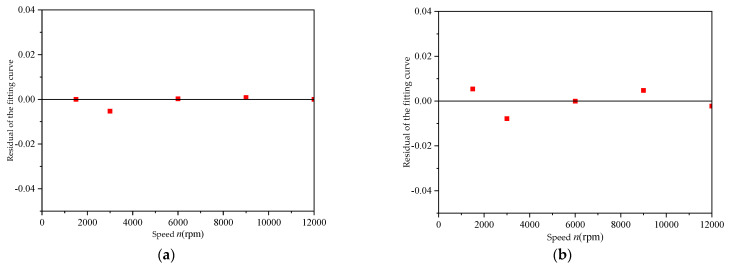
Residuals of different fitting curves: (**a**) exponential function fitting and (**b**) power function fitting.

According to the simulation results, the law of the change of the reduction coefficient with the rotation speed is an exponential function or a power function relationship. Using the least-squares method to fit the relationship between the reduction factor and the rotation speed, the square sum of the errors of the simulation results and the fitting curve calculation results was minimized. The results are shown in Figure 9. By comparing the residual values of the fitted curves and the simulation results under different function relationships (Figure 10), it can be seen that the residual values of the fitted curves were smaller when the exponential function was selected, which indicates that the fitted curves using the exponential function can better characterize the variation of the reduction coefficient from low to high speed. Thus, the formula for obtaining the variation of the reduction coefficient with speed is written as

(11) $$k_{z}=0.15e^{\left(-\frac{n}{3418}\right)}-0.11\;(1500\;\mathrm{rpm}\leq n\leq 12000\;\mathrm{rpm}).$$

**Figure 11:**
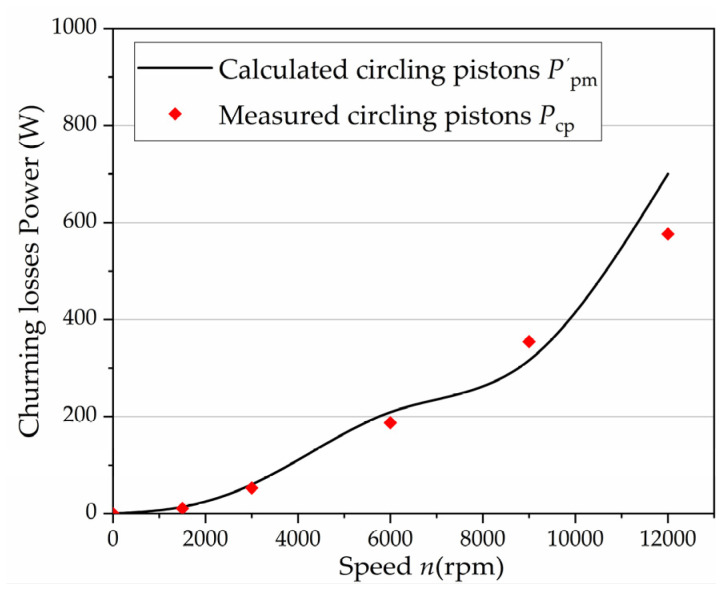
Comparisons between the experimental results and the results from the expression of churning losses with the reduction coefficient for multi-pistons.

Thus, the expression of churning losses due to the multi-pistons from the fitting curve calculation results *P*_pm_’ can be expressed as:

(12) $$P_{\mathrm{pm}}^{’}=\left(1+zk_{z}\right)C_{d}\left\{(\frac{1}{2}\rho \omega^{3}R^{3}d)\sum_{i=0}^{z}\left[l_{0}-R\left(1-\cos\frac{2\pi i}{z}\right)\tan\gamma \right]\right\}$$

where *k*_z_ can be obtained from Equation (11). The results from the method of the expression of churning losses due to the multi-pistons can be made by the above formula, as shown in Figure 11. These curves represent a sample of the results obtained from the many tests and simulations conducted with various speeds in the 0–12,000 rpm range. A satisfactory agreement was observed about the trend of the curve.

## 4. Discussion

**Figure 12:**
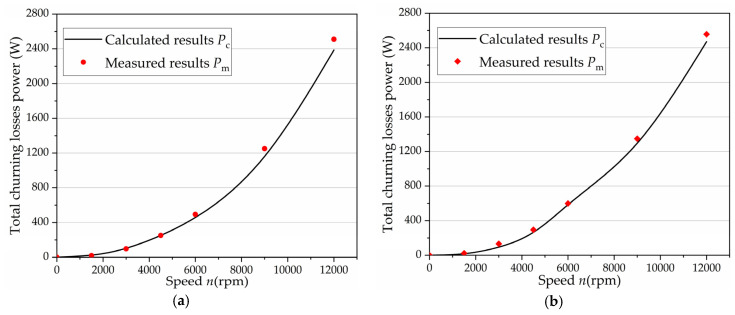
Comparisons between the experimental and calculated results of total churning losses: (**a**) tested churning losses power of nine pistons at different speeds; and (**b**) tested churning losses power of six pistons at different speeds.

As a result of the previous analysis, the theoretical model analysis of the churning losses caused by the cylinder block and pistons were performed separately. The sum of the calculated value of the churning losses caused by the cylinder block and pistons was also verified by experimental measurements on the specific test rig. As shown in Figure 12a, the average error between the test results and the calculated results was less than 10%, which proves that the test results can verify the accuracy of the theoretical model. In addition, the number of pistons was changed in the high-speed axial piston pump in order to verify whether the calculations obtained by the churning losses prediction model could be used for other conditions. Figure 12b shows that the theoretical model can predict the churning losses in different geometries of the axial piston pump. It is known from Reference [3] that cavitation and flow occur around the pistons at high speed, resulting in a reduction coefficient. From the formula, it can be seen that the absolute value of the reduction coefficient increases with the increase of rotating speed and the churning losses of the pistons increase with the increase of the number of pistons. It is known from Reference [3] that the increased churning losses of the pistons are less than those of the cylinder block at high speed due to the cavitation and flow around the pistons. The churning losses power of six pistons was less than that of nine pistons at 1500 rpm and 3000 rpm. Because the churning losses are mainly caused by the pistons at low speed, the increased churning losses of the pistons were higher than that of the cylinder block.

**Figure 13:**
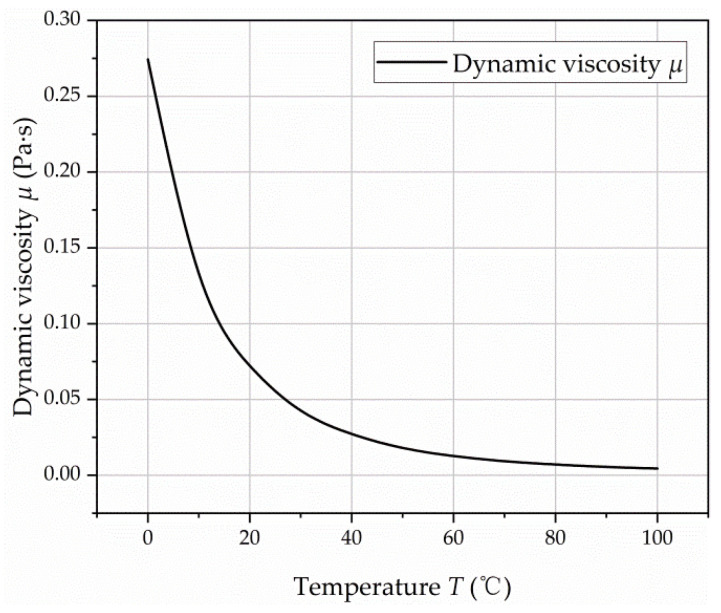
The viscosity of No. 32 hydraulic oil varies with temperature.

**Figure 14:**
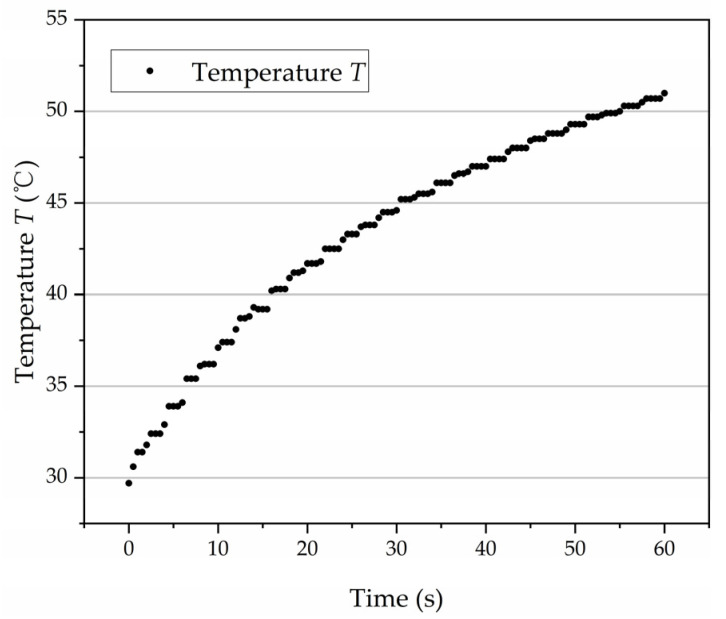
Temperature changes with time at 15,000 rpm.

**Figure 15:**
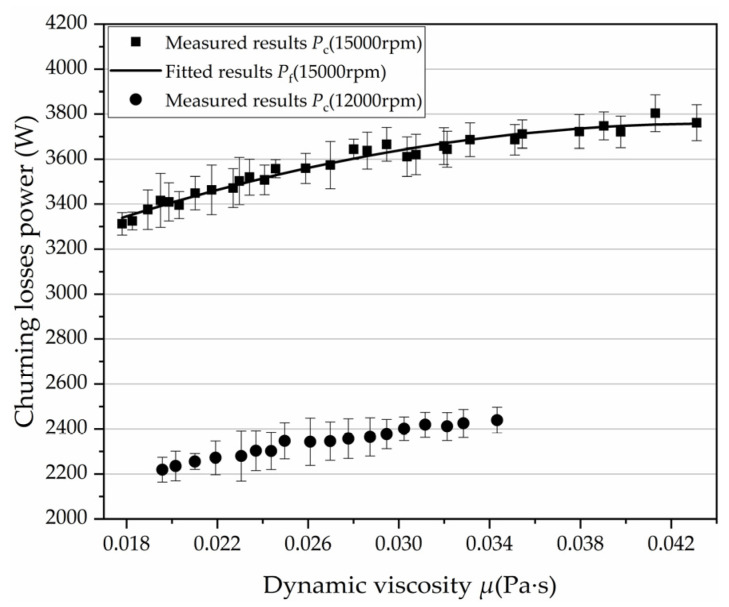
The variation law of churning losses power with oil viscosity.

In this study, the viscosity of oil was indirectly changed by changing the temperature of the oil. No. 32 hydraulic oil was used in the test and the viscosity-temperature characteristic curve was drawn according to Reference [28], as shown in Figure 13. For the effect of the high-speed turbulent flow and low-speed laminar flow on the churning losses, the actual cause was the viscosity change caused by the temperature change. When running at low speed, the temperature change in the test pump was not obvious, so the temperature change by churning measured by the temperature sensor was very small. The large amount of heat generated by the churning losses under high-speed conditions can significantly increase the oil temperature. No cooling or heating was taken during the operation of the axial piston pump, and data were recorded when the speed stabilized at 15,000 rpm. As shown in Figure 14, the heat lost by stirring at 15,000 rpm made the oil temperature rise from 29.7 °C to 51 °C within 1 min. According to the viscosity-temperature characteristic curve in Figure 13, the corresponding viscosity range of 29.7–51 °C is 0.018–0.043 Pa·s. The experimental results of churning losses power under different oil viscosity at 15,000 rpm are shown in Figure 15. The test results show that the churning losses increase with the increase of viscosity, but the growth rate gradually decreases with the increase of viscosity. Through curve fitting, the expression of churning losses as a function of oil viscosity at 15,000 rpm can be obtained, as shown in Equation (13). In addition, by analyzing the test data of 12,000 rpm (Figure 15) and the simulation and test data in Reference [9], it was found that the variation rule of their churning losses with viscosity was the same as that of Equation (13). This proved that Equation (13) has certain reliability under its corresponding conditions.

(13) $$P_{f}=-6.12\times 10^{5}\mu^{2}+5.38\times 10^{4}\mu +2575.03\;(0.018\leq \mu \leq 0.043,15000\;\mathrm{rpm})$$

As shown in Equations (7), (12) and (13), the churning losses can be reduced by modifying structural parameters and reducing oil viscosity and rotational speed. Generally, the oil viscosity and rotational speed are determined by the operating conditions of the axial piston pump, which are not easy to change. Therefore, modification of structural parameters, such as reducing the volume of the stirring oil, can effectively reduce churning losses at high speed.

## 5. Conclusions

(1) This paper proposed an empirical model that can predict the churning losses of axial piston pumps at high speed. The effectiveness of the model was verified by the experimental results of churning loss tests under different geometries and working conditions of the axial piston pump.

(2) The model focuses on the influence of volume and multi-pistons on churning losses. For low-speed conditions, the churning loss is inversely proportional to the volume because the friction losses of laminar viscosity are dominant on churning losses. On the contrary, the churning loss is proportional to the volume in high-speed conditions due to the increase of the oil pulsation. The reduction coefficient is thus decreased and the effect of multi-pistons circling is enhanced with the increase of the rotational speed.

(3) It can be seen from the churning loss model established in this paper that the churning losses can be reduced by changing the structural parameters and the unstructured parameters. In the context of minimizing energy losses, one of the possible ways to reduce churning losses is to optimize the shape of the casing. In addition, this can also reduce oil viscosity and rotational speed.

In view of the continuity of the research topics, in future work, we may study the law of churning losses with temperature and oil viscosity changes under low-speed laminar flow conditions.

## Figures and Tables

**Table 1:** Geometric dimensions and operating conditions of the test pump.

Parameters	Value
The number of pistons *z*	9
The diameter of the piston *d* (m)	0.01
The pitch circle radius of the piston bores *R* (m)	0.02
The radius of the cylinder block *R*_c_ (m)	0.028
The length of the piston out of the cylinder block at the outer dead point *l*_0_ (m)	0.0165
The length of the cylinder block *l*_c_ (m)	0.0325
The gap between the cylinder block and the housing internal surface *t* (m)	0.0145
Test speed *n* (rpm)	1500/3000/6000/9000/12,000
Dynamic viscosity *μ* (Pa·s)	0.0278
Density of the test fluid *ρ* (kg/m^3^)	850

**Table 2:** Reduction Coefficient.

Speed	*k*_z_
1500 rpm	−0.0072
3000 rpm	−0.0471
6000 rpm	−0.0783
9000 rpm	−0.0930
12,000 rpm	−0.1001

## Nomenclature

d	the diameter of the piston (m)
*C*_d_	the drag coefficient
*k*_z_	the reduction coefficient
*l*_0_	the length of the piston out of the cylinder block at the outer dead point (m)
*l*_c_	the length of the cylinder block (m)
*M*_cc_	the experimental churning losses torque due to the rotation of the cylinder (N·m)
*M*_cp_	the experimental churning losses torque due to the circling pistons (N·m)
*M*_dc_	the experimental torque acting on the shaft because of the rotation of the cylinder without oil in the case (N·m)
*M*_dcp_	the experimental torque acting on the shaft because of the circling pistons and the rotating cylinder without oil in the case (N·m)
*M*_wc_	the experimental torque acting on the shaft because of the rotation of the cylinder with oil in the case (N·m)
*M*_wcp_	the experimental torque acting on the shaft because of the circling pistons and the rotating cylinder with oil in the case (N·m)
n	the test speed (rpm)
*P*_cc_	the experimental churning losses power due to the rotation of the cylinder (W)
*P*_cm_	the calculated churning losses power due to the rotation of the cylinder (W)
*P’*_cm_	the calculated churning losses power due to the rotation of the cylinder after optimization (W)
*P*_cp_	the experimental churning losses power due to circling pistons (W)
*P*_pm_	the calculated churning losses power due to circling pistons (W)
*P’*_pm_	the calculated churning losses power due to circling pistons after optimization (W)
R	the pitch circle radius of piston bores (m)
R_c_	the radius of the cylinder block (m)
Re	the Reynolds number
t	the gap between the cylinder block and the housing internal surface(m)
V	the volume of the stirring oil (m^3^)
z	the number of pistons
ω	the rotation angular velocity (rad/s)
μ	the dynamic viscosity (Pa·s)
ρ	the density of test fluid (kg/m^3^)
γ	the swash plate angle (rad)
